# Supplementary material for: Effective La-Na Co-Doped TiO2 Nano-Particles for Dye Adsorption: Synthesis, Characterization and Study on Adsorption Kinetics
Source: Nanomaterials (Basel). 2019 Mar 9;9(3):400. doi: 10.3390/nano9030400 (PMC6474146; doi:10.3390/nano9030400)
Supplement: Supplementary file 1 [file nanomaterials-09-00400-s001.pdf]

Supplementary File

Effective La-Na co-doped TiO_2 nano-particles adsorbent for dye removal: Synthesis, characterization and study on adsorption kinetics.

Inderjeet Singh and Balaji Birajdar*

Special Centre for Nano Sciences, Jawaharlal Nehru University, New Delhi-110067, India

Corresponding Author: *birajdar@mail.jnu.ac.in*

Corresponding Author, Tel: +91 112670474

Figure caption:

Fig. S1 High resolution XPS spectra of Ti 2p of prepared samples.

Fig. S2 High resolution XPS spectra of La 3d of prepared samples.

Fig. S3 High resolution XPS spectra of Na 1s of prepared samples.

Fig. S4 (a) Effect of pH on absorbance spectra of MB dye. **(b)** Amplified image of (a) at pH 7 and pH 9.

Table caption:

Table S1 Area under different peaks obtained from high resolution XPS of different samples.

Table S2 FWHM of different peaks obtained from high resolution XPS of different samples.

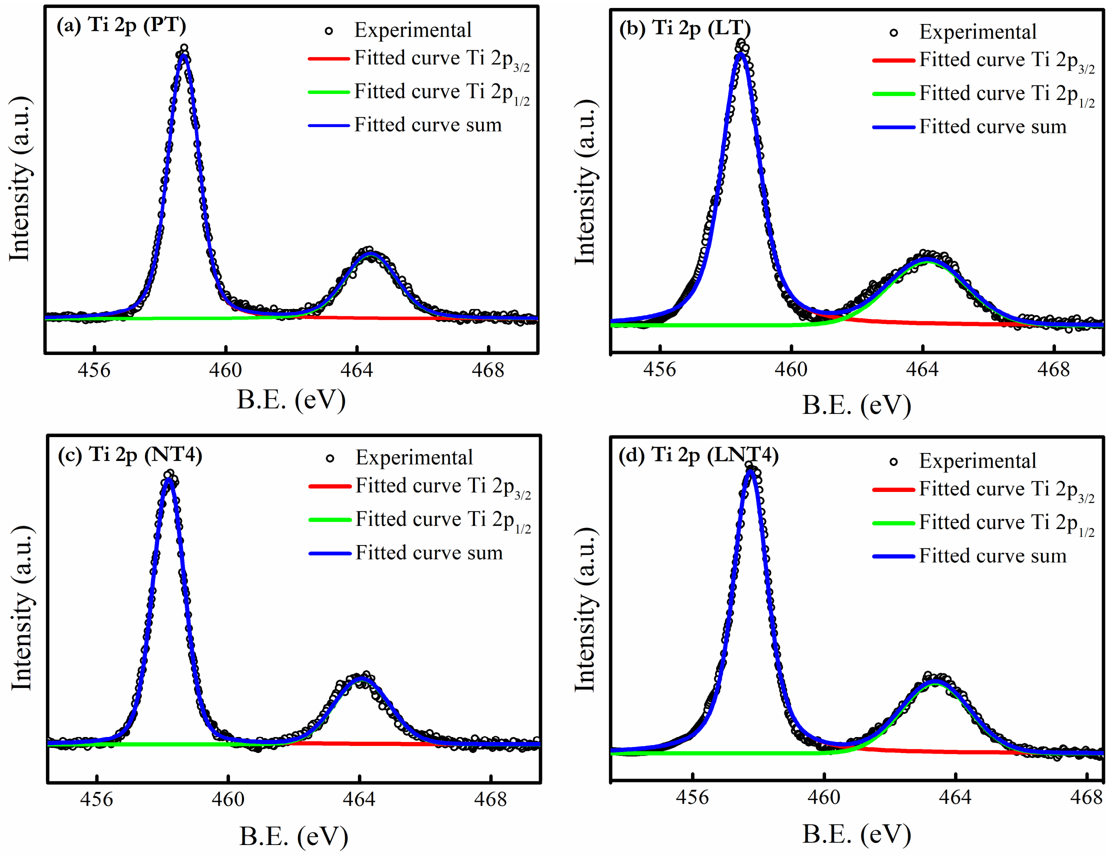

Fig. S1 High resolution XPS spectra of Ti 2p.

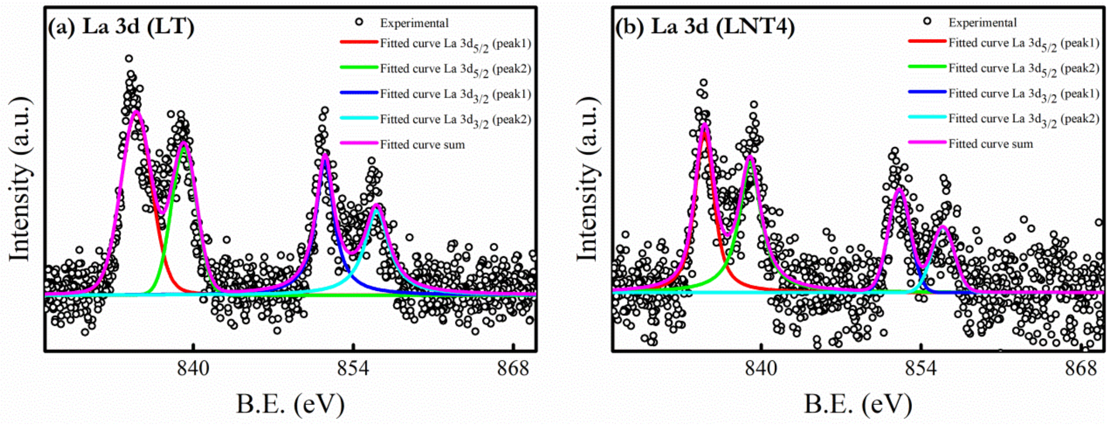

Fig. S2 High resolution XPS spectra of La 3d.

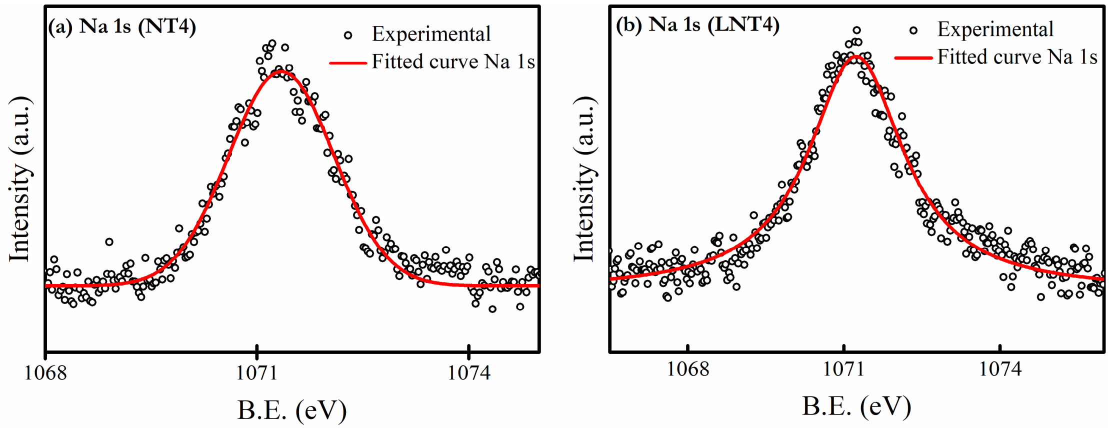

Fig. S3 High resolution XPS spectra of Na 1s.

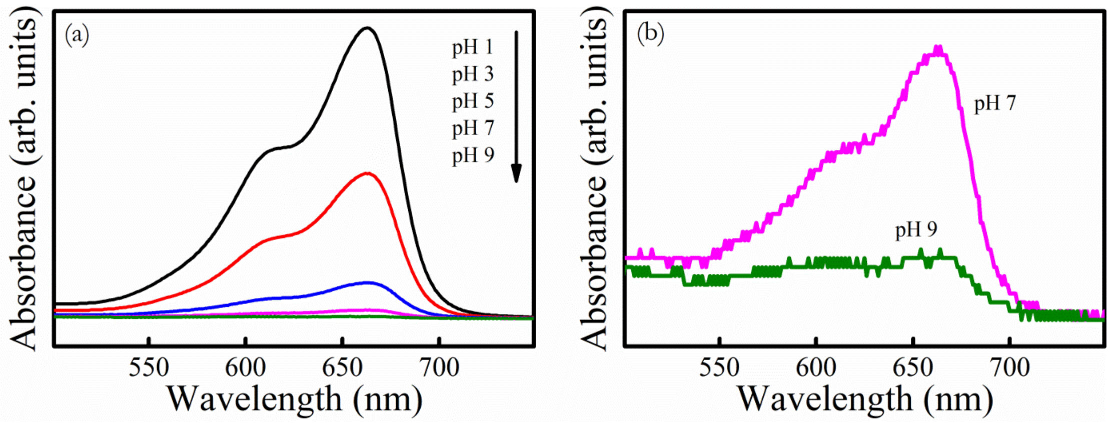

Fig. S4 (a) Effect of pH on absorbance spectra of MB dye. **(b)** Amplified image of (a) at pH 7 and pH 9.

Table S1 Area under different peaks obtained from high resolution XPS of different samples.

Sample	Area under								
	Ti-O peak	O-H peak	Ti $2p_{3/2}$ peak	Ti $2p_{1/2}$ peak	La $3d_{5/2}$		La $3d_{3/2}$		Na 1s peak
					Peak 1	Peak 2	Peak 1	Peak 2	
PT	8787.77	2526.69	6452.93	2576.94	-	-	-	-	-
LT	8921.57	3991.99	6362.42	2475.54	2782.12	1734.34	1733.14	1456.75	-
NT4	9334.62	4126.57	5770.72	2280.54	-	-	-	-	2452.25
LNT4	9716.55	4273.79	5535.63	2119.75	1861.97	1310.98	696.09	459.29	1628.04

Table S2 FWHM of different peaks obtained from high resolution XPS of different samples.

Sample	FWHM								
	Ti-O peak	O-H peak	Ti $2p_{3/2}$ peak	Ti $2p_{1/2}$ peak	La $3d_{5/2}$		La $3d_{3/2}$		Na 1s peak
					Peak 1	Peak 2	Peak 1	Peak 2	
PT	1.23	2.44	1.13	1.92	-	-	-	-	-
LT	1.22	2.45	1.21	2.15	3.21	2.64	1.96	2.61	-
NT4	1.24	2.64	1.14	2.01					2.23
LNT4	1.20	1.86	1.20	2.12	2.03	2.32	1.85	2.36	1.77
